# On the Applicability of Elastic Network Models for the Study of RNA CUG Trinucleotide Repeat Overexpansion

**DOI:** 10.1371/journal.pone.0152049

**Published:** 2016-03-24

**Authors:** Àlex L. González, Jordi Teixidó, José I. Borrell, Roger Estrada-Tejedor

**Affiliations:** Grup d’Enginyeria Molecular (GEM), Institut Químic de Sarrià (IQS) – Universitat Ramon Llull (URL), Barcelona, Catalonia, 08017, Spain; University of Texas MD Anderson Cancer Center, UNITED STATES

## Abstract

Non-coding RNAs play a pivotal role in a number of diseases promoting an aberrant sequestration of nuclear RNA-binding proteins. In the particular case of myotonic dystrophy type 1 (DM1), a multisystemic autosomal dominant disease, the formation of large non-coding CUG repeats set up long-tract hairpins able to bind muscleblind-like proteins (MBNL), which trigger the deregulation of several splicing events such as cardiac troponin T (*cTNT*) and insulin receptor’s, among others. Evidence suggests that conformational changes in RNA are determinant for the recognition and binding of splicing proteins, molecular modeling simulations can attempt to shed light on the structural diversity of CUG repeats and to understand their pathogenic mechanisms. Molecular dynamics (MD) are widely used to obtain accurate results at atomistic level, despite being very time consuming, and they contrast with fast but simplified coarse-grained methods such as Elastic Network Model (ENM). In this paper, we assess the application of ENM (traditionally applied on proteins) for studying the conformational space of CUG repeats and compare it to conventional and accelerated MD conformational sampling. Overall, the results provided here reveal that ANM can provide useful insights into dynamic rCUG structures at a global level, and that their dynamics depend on both backbone and nucleobase fluctuations. On the other hand, ANM fail to describe local U-U dynamics of the rCUG system, which require more computationally expensive methods such as MD. Given that several limitations are inherent to both methods, we discuss here the usefulness of the current theoretical approaches for studying highly dynamic RNA systems such as CUG trinucleotide repeat overexpansions.

## Introduction

Many biological processes involve concerted interactions of macromolecules, such as protein-protein or protein-nucleic acids complexes. For this reason, the role of such dynamics mechanisms is increasingly important. Learning about how biomolecular interactions activate these biological processes may help us to get a better understanding of the underlying causes of diseases and improve drug design strategies to modulate and optimize ligand-macromolecule interactions. Functional motions of proteins have been widely explored but of the dynamic behavior of RNA has only recently been addressed. Modeling RNA flexibility remains extremely challenging, owing to the complexity of the conformational landscape of this type of macromolecule.

It is becoming increasingly common to study nucleic acids structures for the design of small molecules or peptides that target a particular secondary structure, such as microRNA associated with cancer [1,2] or HIV TAR viral RNA [3–6]. RNA-mediated diseases are usually related with non-coding repeat expansions which induce cytotoxicity through different mechanisms.[7–13] Among these, one of the most well-studied diseases involving toxic RNA is myotonic dystrophy (DM).[14,15] DM is an inherited multisystemic disease which affects the skeletal and smooth muscle, the eyes, the heart, the endocrine system and central nervous system. At the molecular level, DM involves an aberrant CTG trinucleotide expansion in the *DMPK* gene (which induces myotonic dystrophy type 1, DM1)[15–17] or a CCTG tetranucleotide expansion in the intron 1 of the *CNBP* gene (myotonic dystrophy type 2, DM2)[18–20]. Subsequent pre-mRNA microsatellites formed during transcription contain expanded CUG or CCUG repeats (henceforth named rCUG and rCCUG respectively) which are capable of sequestering nuclear RNA-binding proteins involved in splicing events such as the ones in the muscleblind-like family (MBNL).[21–23] Consequently, these proteins are deprived of their normal functions and induce RNA foci formations. [19,24]

Characterization of the pathogenic transcript secondary structure is crucial to gain insights into the DM pathogenic pathway. For instance, rCUG sequences are usually considered as long tract hairpins, but experimental evidence has demonstrated that rCUG microsatellites fold into metastable hairpins with a variety of secondary structures that directly determine protein-binding properties.[25] UV melting [26] and nuclease mapping [27] studies have revealed that rCUG stability is mainly driven by the A-form geometry given by the C•G and G•C canonical base-pairs formation, which increases with the hairpin length. Additionally, X-ray crystallography [28–33] and NMR [34] have demonstrated that 1×1 internal loops formed by U-U pairs are quite loose and capable of adopting a variety of conformations involving a different number of hydrogen-bonds; however, other studies have concluded that in CUG, CCG and CAG repeats, higher hydrogen bond-forming potential does not result in higher stability.[26] Some studies successfully have performed and analyzed the dynamics of different pathogenic transcripts using molecular dynamics (MD), proving the ability of this technique to correctly describe RNA dynamic-function relationships.[34–36] The relative population analysis of the U-U pairs revealed that these mismatches are able to explore different conformations involving up to two hydrogen bonds without distorting the A-form geometry, leading to results that are in good agreement with NMR and X-ray data.

Unfortunately, for the time being, MD requires thousands of hours and usually demands supercomputing resources. Crucial to the usefulness of MD methods are the reliability of RNA force fields because their parameterization is still imprecise and may impact both small and large-scale dynamics. In contrast, elastic network models (ENM) allow for the exploration of the conformational dynamics of a molecular system near its equilibrium conformation using a harmonic potential function.[37] Interestingly, though ENM has been widely used on proteins, [38–43] its application on RNA folded structures has only recently been reported.[44,45] The advantage of ENM relies on reducing the macromolecule structure to a network of nodes interconnected by springs. The dynamics if the molecular system can then be described by monitoring of the network. In particular, anisotropic network models (ANM), [46] one of the most widely used ENM strategies, establish the node connectivity according to a cutoff distance (r_c_) and define springs with uniform force constants (γ). Nonetheless, although the usefulness of these methods has been proved, ENM do not consider any type of interaction between pairs of atoms. Furthermore, the dynamics rely only on the proximity of the defined nodes. In addition, ENM neglect nonlinear effects or couplings between nodes and do not take into account nor solvent effects neither ion distribution around the substrate, which may be essential for understanding internal loop local motions. On one hand, thermally accessible conformational substates take considerable amounts of time to equilibrate in MD simulations; on the other hand, ENM may only capture the near-equilibrium deformability space due to the inexistence of potential energy barriers between substates.

Despite the inherent limitations of these methods, previous studies have shown that ENM are able to describe experimentally observed RNA’s backbone conformational movements.[44,45] Within this context, we sought to investigate the capability of ANM and MD to reproduce appropriately the structural dynamics of the repeat-associated pathogenic transcript rCUG. First, we investigated the intrinsic dynamics of experimental rCUG repeats using ENM techniques (see *ANM of the rCUG ensemble* section) and compared them to the ones obtained from a conventional molecular dynamics (cMD) simulation (as shown in Conventional MD of the rCUG system model). Ensembles from either macromolecules or MD trajectories can be analyzed using essential dynamics analysis (EDA). EDA is based on the principal component analysis (PCA) of the covariance matrix. Principal components (PCs) can be viewed as directional vectors of the structural variations through space spanned by the principal modes of motion. Similarly, ANM are based on the diagonalization of the Hessian matrix and describe the directions responsible for the movement of the network’s nodes. Thus, we used PCs and ANM results to compare the structural sampling obtained by MD and ANM correspondingly.

Although ANM depend on the number of available experimental structures in order to obtain meaningful results, MD simulations are subject to the sampling problem of the conformational space.[47] We tried to address the MD sampling limitations by using accelerated molecular dynamics (aMD), which provide access to events beyond those obtained with conventional MD (described in Accelerated MD of the rCUG system model). Finally, we discuss the conformational space obtained by all these methods and compare them to those conformers obtained by deformation of the ANM softest normal modes (Comparison of ANM, PCA and EDA modes).

## Results

RNA hairpins containing *N*×*N* internal loops are involved in many diseases and they have caught the attention of drug designers. On the one hand, RNA does not have clear pockets and cavities, like the ones observed in proteins, hampering efforts to achieve selectivity. On the other hand, many investigators have provided evidence that repeated internal loops (e.g. rCUG) can be used as unique anchoring points so that molecules which contain repeated subunits with a spacer achieve better selectivity values. It is therefore clear that a good description of (i) the structure of the internal loops and (ii) the intrinsic dynamics of the nucleotides involved would improve the molecular design of RNA targeted drugs.

Computational methods for predicting the motions of proteins have been thoroughly applied, but the dynamics of many RNA relevant structures still remains elusive. However, MD require extensive computational resources and are subjected to force field limitations. In this study, we investigated the dynamics of a pre-mRNA transcript implicated in an RNA-mediated disease, specifically DM1 (trinucleotide CUG repeats, rCUG) using two distinct computational methods to represent its intrinsic flexibility; these were elastic network models (ENM) and MD.

### ANM of the rCUG ensemble

Anisotropic network models (ANM)[46] are based on the assumption that a system can be described as particles connected by springs. Therefore, the intrinsic flexibility and dynamics of the macromolecule are treated as a set of normal modes within an oscillating system. Recent studies have assessed the viability of ANM for conveying a set of apparent motions in RNA and DNA ensembles and have succeeded in reproducing those observed in experimental data.[44,45,48] By following these approaches, we sought to investigate a particularly relevant RNA structure such as CUG trinucleotide repeat overexpansion.

Many RNA structures that include one or more CUG triplets in their sequence have been reported by different investigators. We considered static X-ray dataset constituted an ensemble of snapshots representative of the intrinsic dynamics of the RNA. The ensemble can be analyzed using principal component analysis (PCA) to extract the principal modes of structural variations (i.e. principal component, PC) which, in turn, can be compared to ANM soft modes. Nevertheless, the number and type of RNA motions are subject to the number of conformations that have been experimentally resolved.

**Fig 1:**
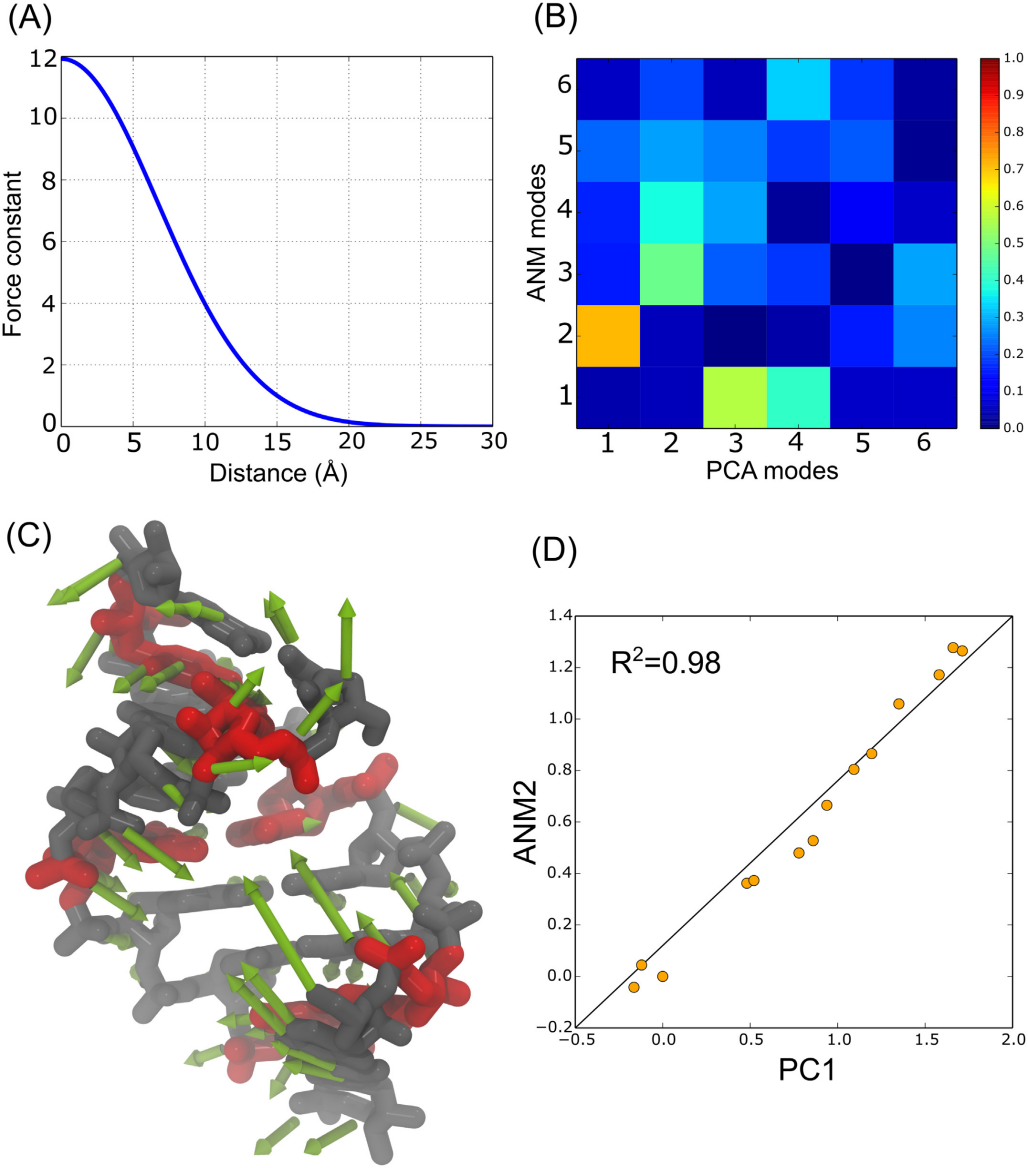
(A) Optimized distance dependent force constant (γ). Closest nodes are weighted by 12 (arbitrary units), and the weighting decays exponentially down to 25 Å. (B) Overlap between the top six PCA modes and the softest six NMA modes. The second softest ANM mode exhibits the highest overlap with PC1. (C) All-atom representation of (CUG)_3_ fragment and PC1 normal mode vectors. U-U pairs are represented in red. Normal mode vectors (in green) show the structural variations along this mode. (D) Representation of the dispersion of the examined PDB structures along the PC1 and ANM2.

The available structural models of rCUG transcripts in the RCSB Protein Data Bank should be sufficient for dynamics simulations using PCA, although these models are quite sensitive to small changes in structure. We performed the PCA on a collection of rCUG structures with a number of repeats ranging from 3 to 6. First, a (CUG)_3_ ensemble was constructed by aligning all the possible (CUG)_3_ fragments from the PDB X-ray structures (see details in Methods). Then, the ability of ANM to capture variations within the structural ensemble was evaluated. Previous studies suggested that the inclusion of ribose atoms into the coarse-grained model improved the level of experimental agreement compared to the use of only phosphorous atoms.[44] Following these criteria, we decided to test an all-atom model and two levels of coarse-graining: P, C2’, C4’ atoms, and P, C2’, C4 and N3 atoms. We considered it important to capture the hydrogen-bonding patterns and the U-U mobility, thus we sought to capture this information through the N3 and C4 atom while P and C2’ describe the backbone dynamics. At the same time, γ constant was optimized using a negative exponent weighting approach (refer to Fig 1A). Different cutoffs ranging from 5 to 15 Å were tested. Finally, the generated ANM models were compared to PCs obtained from the PCA of the ensemble.

The best ANM model was achieved using a cutoff of 9 Å and a coarse-grained model represented by atoms P, C2’, C4 and N3. No improvement was achieved by using an all-atom model in terms of overlapping modes (see S1 Fig); hence, some loss of description level have occurred even though the essential motions of the RNA are correctly described. When comparing ANM modes with PCs we observed that the second NMA mode exhibits an acceptable overlap with PC1 mode (71%), see Fig 1B. In other words, the second softest ANM theoretical mode is confirmed by the first experimental PC.

Visual inspection of the dominant motions extracted from coarse-grained PCA shows that PC1 deformation vector corresponds to the bending of the RNA structure from end to end, opening and closing the major groove (Fig 1C). Moreover, PC1 and ANM2 show an excellent correlation (0.98), as shown in Fig 1D, meaning that the crystal structures have low dispersion around this pair of PCA and ANM modes. Interestingly, PC2 and PC3 represent the movement of the base pairs along the *xy* plane. We observed that C•G and G•C pairs shear in opposite directions along the plane, but U-U pairs move cooperatively in the same direction exhibiting a base pair opening. Compared to the backbone movement, these modes overlap with the theoretical modes by 48% and 57% respectively.

**Fig 2:**
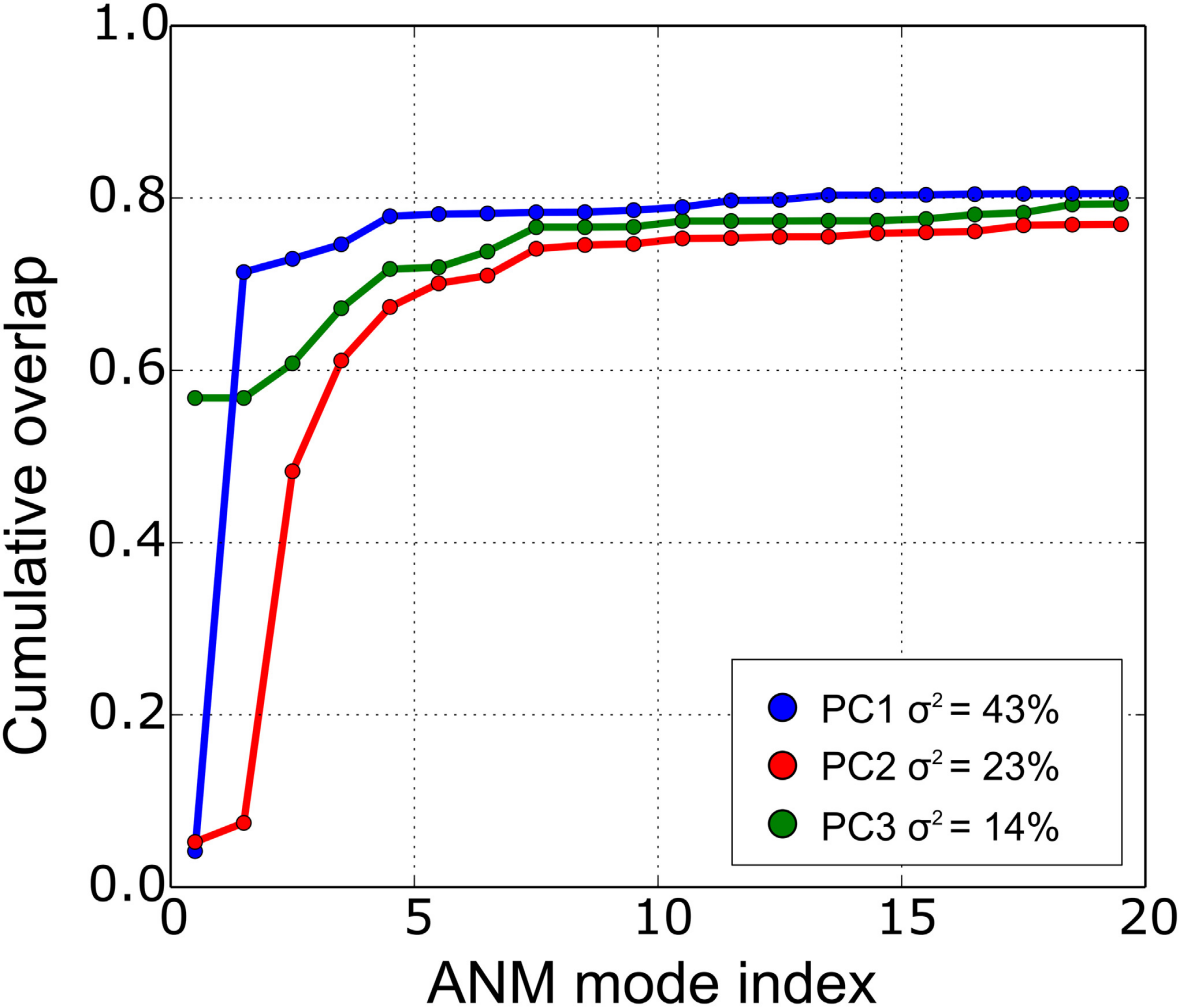
Cumulative overlap of ANM soft modes to PC1 to PC3. The legend contains the percentage of variance (σ^2^) explained by the corresponding PC. Notice that 20 ANM modes explain ~80% of structural variations along the first and third PCs.

The principal structural changes can be described through a set of low-frequency ANM modes. The cumulative overlap can be interpreted as the extent to which this set of soft modes can predict a PCA mode. In Fig 2, we report the cumulative overlap of the first three PC and the percentage of captured variance. Notice that 20 ANM modes can explain ~80% of PC1 and PC3. In fact, the ANM1-12 provide a good description, while higher modes diminish its contribution. Nonetheless, the distribution of motions is captured by the collectivity degree (κ) which provides a measure of the extent of distribution of motions across the structure. The collectivity degrees for the first three PCs are 0.43, 0.64 and 0.74 correspondingly, meaning that the first PC motions are distributed less frequently along the structure. In fact, PC2 and PC3 are mainly represented by the shear and stretching of all the base pairs, especially of the U-U internal loops; thus, these two modes are considered to be highly collective and more relevant for the present study.

### Conventional MD of the rCUG system model

In good agreement with previous reports, our approach shows that ANM can achieve an acceptable level of description of global RNA dynamics. However, ANM are not able to correctly describe local dynamics and the available set of structures do not necessary represents all accessible structural changes. For this reason, further rCUG dynamics were assessed using conventional MD (cMD) simulations. All the MD analysis was performed according to the observed U-U conformations along the trajectory, which are the most relevant local changes, and compared to the experimentally resolved structures.

Kiliszek *et al*. noticed that some uridines involved in U-U pairs tilt towards the minor groove, breaking the palindromic symmetry in a seemingly random manner over the structure.[29] The number of CUG repeats determines the number of available three-dimensional U-U structures and hence, in longer RNA chains, there must exist a vast repertoire of U-U pair in terms of available conformations. Kumar *et al*. reported two rCUG X-ray structures providing different 1×1 nucleotide U-U internal loop conformations and considered that the small molecule drug design should take into account all the available U-U conformations.[32]

**Fig 3:**
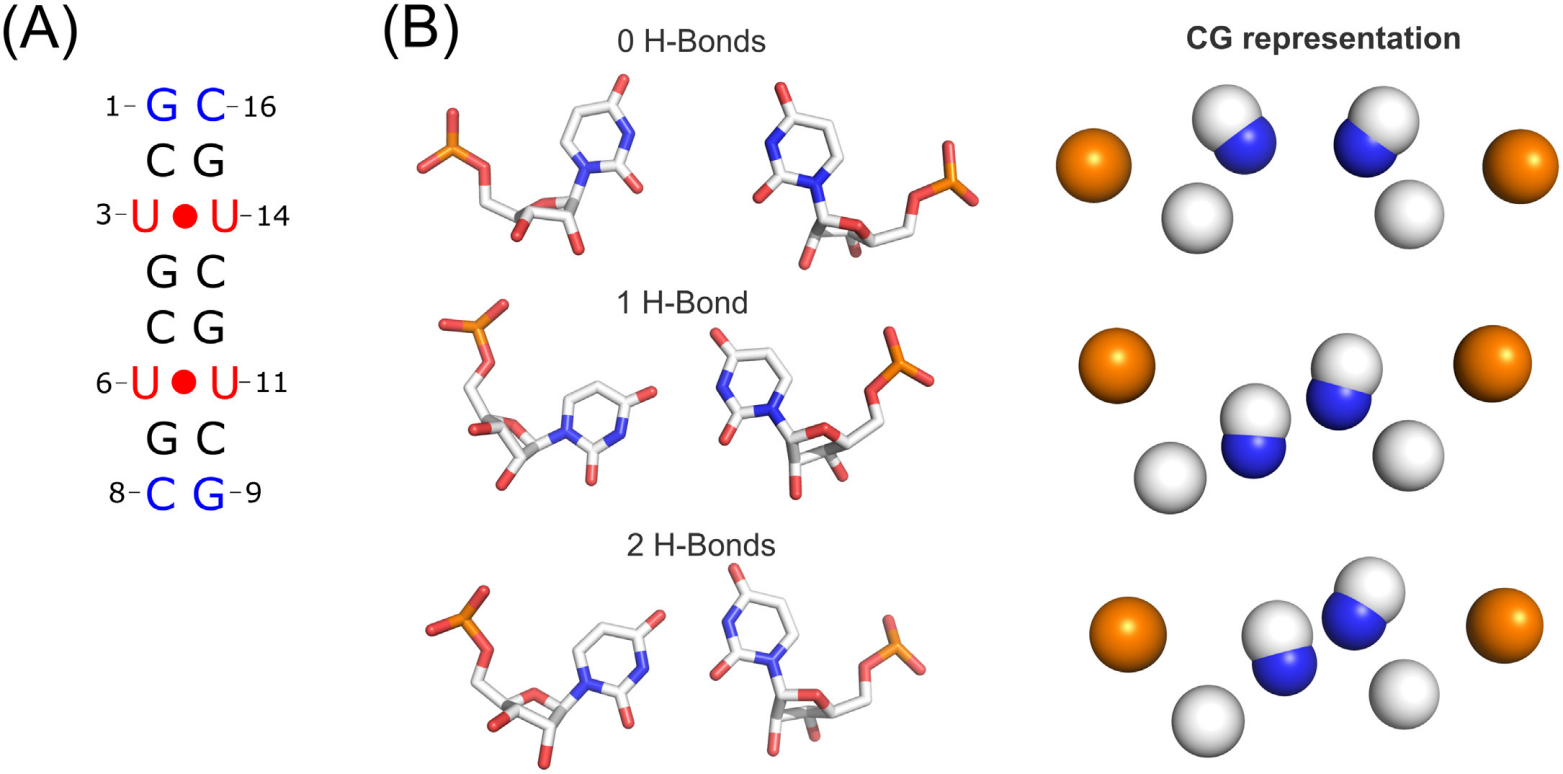
(A) Schematic representation of the system model used for the cMD. (B) Representative U-U pair types observed along the trajectory (left) and coarse-grained (CG) schematic representation (right). White, red and blue spheres represent selected carbon, oxygen and nitrogen beads respectively.

Here we studied the behavior of the rCUG model through cMD simulations and determined the dynamic properties of these particular non-canonical U-U pairs. Our model system consists of two CUG repeats capped by C•G pairs, to increase the overall stability during the simulation (see Fig 3A). The non-canonical base-pairs of the system model adopt the stretched U-U wobble conformation; this conformation establishes interactions with only one hydrogen bond between the carbonyl O4 and the N3 imino group of the second residue.

After the cMD simulation we identified a total of 4 possible U-U pair conformations by means of cluster analysis, which featured different hydrogen bonding patterns. Among the most representative conformations, we observed the presence of 0, 1 or 2 direct hydrogen bonds, some of which formed 1 or 2 water-mediated hydrogen bonds at the same time. The population of U-U clustered conformations for each base pair are reported in the Supporting Information. During the simulation each pair went through nearly two or three distinct conformations, although some of them lasted for less than 5% of simulation time (Fig 3B). From highest to lowest population, MD results suggest that U-U pairing formation involves 1, 0 or 2 hydrogen bonds, whereas water mediated hydrogen bonds represent a 20% of total simulation time. The analysis of the overall structure along the MD trajectory revealed that only the helical opening was statistically significant, at the 95% confidence level, compared to the crystal structure. All of these hydrogen bond patterns have been reported in a combined NMR and MD study of (CCGCUGCGG)_2_.[34] In good agreement with our results, Parkesh *et al*. reported that after sampling all the possibilities, the lowest-energy motif presented a single hydrogen bond.

Further non-canonical U-U pair structural analysis was performed in accordance with Berglund and coworkers’ observations.[33] The authors noticed that six U-U conformations were present in all X-ray and NMR available structure, if classified by their number of hydrogen bonds and inclination (not inclined, or inclined towards the major or minor groove). These types of U-U pairs are named following previously established criteria: type I non-canonical U-U pairs contains 2 hydrogen bonds, with a shortened C1’-C1’ distance and inclined towards the minor groove. Type II forms 1 hydrogen bonds and is also inclined towards the minor groove. Type III do not state significant inclination and do not form any hydrogen bond. The most frequently observed conformation is type IV that forms 1 hydrogen bond and inclines towards the major groove. Types V and VI also inclines towards the major groove but contains 2 or 0 hydrogen bonds respectively. Using this description, we performed a classification using the average structure of the MD trajectory to reproduce the most relevant conformation for each pair.

A qualitative analysis suggested that each of the two average U-U pairs stayed in type IV and II configurations during the simulation. The predominance of these substates has been thoroughly discussed in literature and types IV and II are the most predominant configurations among the crystal structures. Through examination of all rCUG NMR and crystal structures, it becomes clear that U-U pairs can flex between many different conformations. Nevertheless, looking at our MD results (S1 Table) we observed very close C1’-C1’ distances, helical averages and standard deviation with experimental data, which suggest that our MD trajectory was able to explore the most relevant experimentally observed U-U conformations, but not all of them.

### Accelerated MD of the rCUG system model

cMD allow us to reach time scales in the order of hundreds of nanoseconds. However, advanced sampling techniques have been developed to explore structural changes in shorter time scales (e.g. replica-exchange molecular dynamics, metadynamics and accelerated molecular dynamics, among others). Accelerated molecular dynamics (aMD) is an enhanced conformational sampling technique that provides access to events beyond the ones obtained by conventional simulations. For instance, our c MD was run on a sub-microsecond scale and it was not able to explore all the available U-U configuration space, so it became clear that a more exhaustive exploration was required. We decided to assess whether enhanced sampling techniques such as aMD conformational sampling improved the results yielded by cMD.

**Fig 4:**
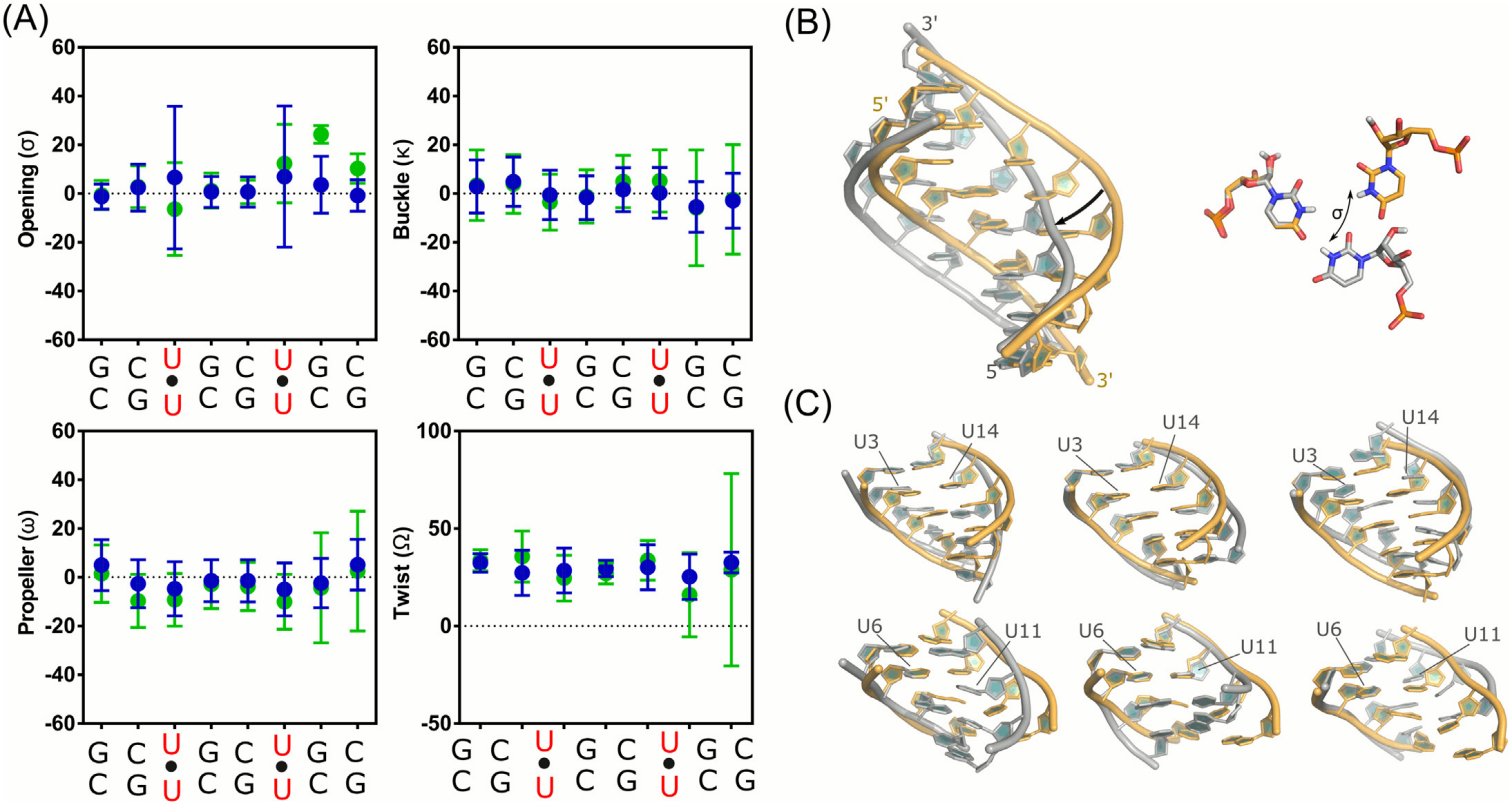
Structural analysis of the cMD and aMD simulations. (A) Average and standard deviation for each helical parameter (base pair opening, buckle, propeller and helical twist). cMD and aMD results are colored in blue and green respectively. Structures were aligned to all heavy atoms and represented with PyMOL. Notice that the 3’-end from the aMD simulation yields significant deviation from the cMD. (B) Cartoon representation of two clustered structures from the cMD simulation and opening effect (σ) of the U-U pair. The two main principal motions observed along the simulation correspond to the backbone expansion-compression (opening and closing of the major groove) and the base pair opening of the uridines. (C) Cartoon representation of the first and second CUG fragments from the aMD simulation. The main distortions are observed in the 3’-end, as stated by the helical parameter values.

Surprisingly, the aMD trajectory tends to drift away from that observed in cMD. As shown in Fig 4 (see also S2 and S3 Figs), helical parameters from both simulation are within the same range but large deviations occur in the 3’-end of the aMD without affecting the second U-U pair. This effect was observed in less than 15% of the simulation (see S4 Fig). This effect might be a force field artifact caused by improper description of non-bonded interactions or backbone definition. In sharp contrast with the cMD results, the aMD trajectory samples a completely different region of the U-U conformational space. For instance, types I, III and V are preferred along the simulations. In both cases, the main motions are governed by the backbone heavy atoms displacement and the base pair opening. This observation agrees with the previous PCA so further comparison with essential dynamics analysis (EDA) was accomplished.

### Comparison of ANM, PCA and EDA modes

Once the MD simulations were analyzed, we proceeded to compare the EDA of the generated trajectories with the *reference* modes obtained from the crystal structures. A previous report benchmarked the sampling of MD protein simulations against ANM and PCA using 20 ns trajectories.[49] Those authors concluded that generating conformers using the softest ANM modes covered a more comprehensive subspace than the MD ensembles. Moreover, ANM requires very low computational resources compared to conventional methods.

**Fig 5:**
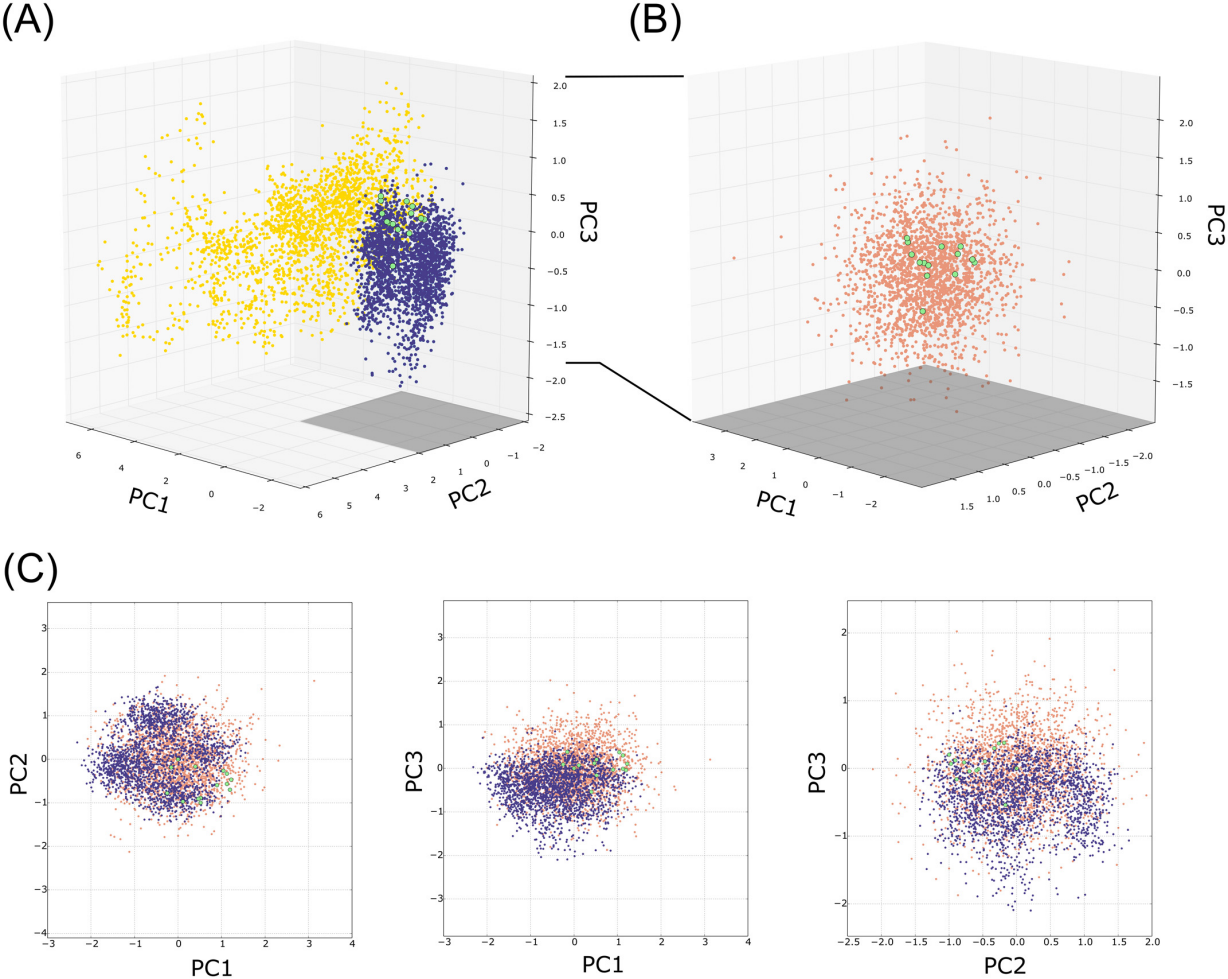
Projection over the PC1-3 subspace of (A) the cMD (blue) and aMD (yellow) snapshots, and the PDB ensemble (green). (B) Comparison between the original PDB ensemble and 2000 conformers (salmon) generated using the softest three modes. The perspective is the same in both panels, but the ranges differ. (C) Two-dimensional projection of the aMD, ANM generated ensemble and PDB ensemble over the first three PCs.

In this study, we asked whether RNA small systems might span similar conformational coverage using any of the aforementioned methods. That is to say, we compared the conformational sampling of experimental structures and simulations, all projected onto the principal subspace spanned by the first three PCs. We represented each ensemble (the PDB ensemble and the cMD and aMD trajectory snapshots) onto the PC1-3 subspace (Fig 5A).

In agreement with our previous analysis of the *reference* or experimental modes, it is clear that the highest collective modes are PC2 and PC3. Compared to PC1, changes are less localized and less pronounced along the other two PCs. In agreement with previous studies, [49–50] conformers generated during cMD and aMD simulations encompass only part of the crystal structure and explores the surrounding subspace. For instance, cMD and aMD conformers drift away from the *references* and reproduce only half of the experimental structures, which is reflected in its low essential space overlap (50% and 49% respectively). In sharp contrast, the essential subspace overlap between cMD and aMD simulations account for a total of 77% of subspace overlap. It is unclear whether the explored aMD conformers correlate events which occur on longer time scales. However, the initial and final aMD frames are locate in the same subspace region, meaning that the structure ‘visits’ several substates but is capable of returning to the initial structure. From a structural point of view, MD simulations span a wide range of U-U base pairs, but also the transition between them and the different combinations of the possible base pairing types. Likewise, the collectivity of the principal modes that describe these motions suggest cooperative dynamics along the structure (see Table 1).

**Table 1:** Variance (σ^2^) and collectivity (κ) for each ensemble system: PDB ensemble and molecular dynamics (cMD and aMD).

	PDB ensemble		cMD		aMD	
Mode	%σ^2^	κ	%σ^2^	κ	%σ^2^	κ
**1**	43.2	0.43	32.7	0.39	25.9	0.22
**2**	22.9	0.64	24.4	0.63	17.4	0.50
**3**	13.89	0.74	11.6	0.73	11.2	0.70

A previous work showed a remarkable coverage of the reference space by ANM predictions. For this reason, we generated 2000 snapshots by deforming the structure along the softest three ANM modes and compared the subspace coverage with that of the cMD. As reported by Bakan and Bahar, [49] deformation along the ANM modes exhibit an excellent coverage of the *references*. Superposition of our generated conformers with the *references* (Fig 5B) show that the ANM modes permit the exploration of a wider range of conformations along the modes. However, we noticed that the first principal mode was strongly favored without a significant loss in terms of collectivity.

**Fig 6:**
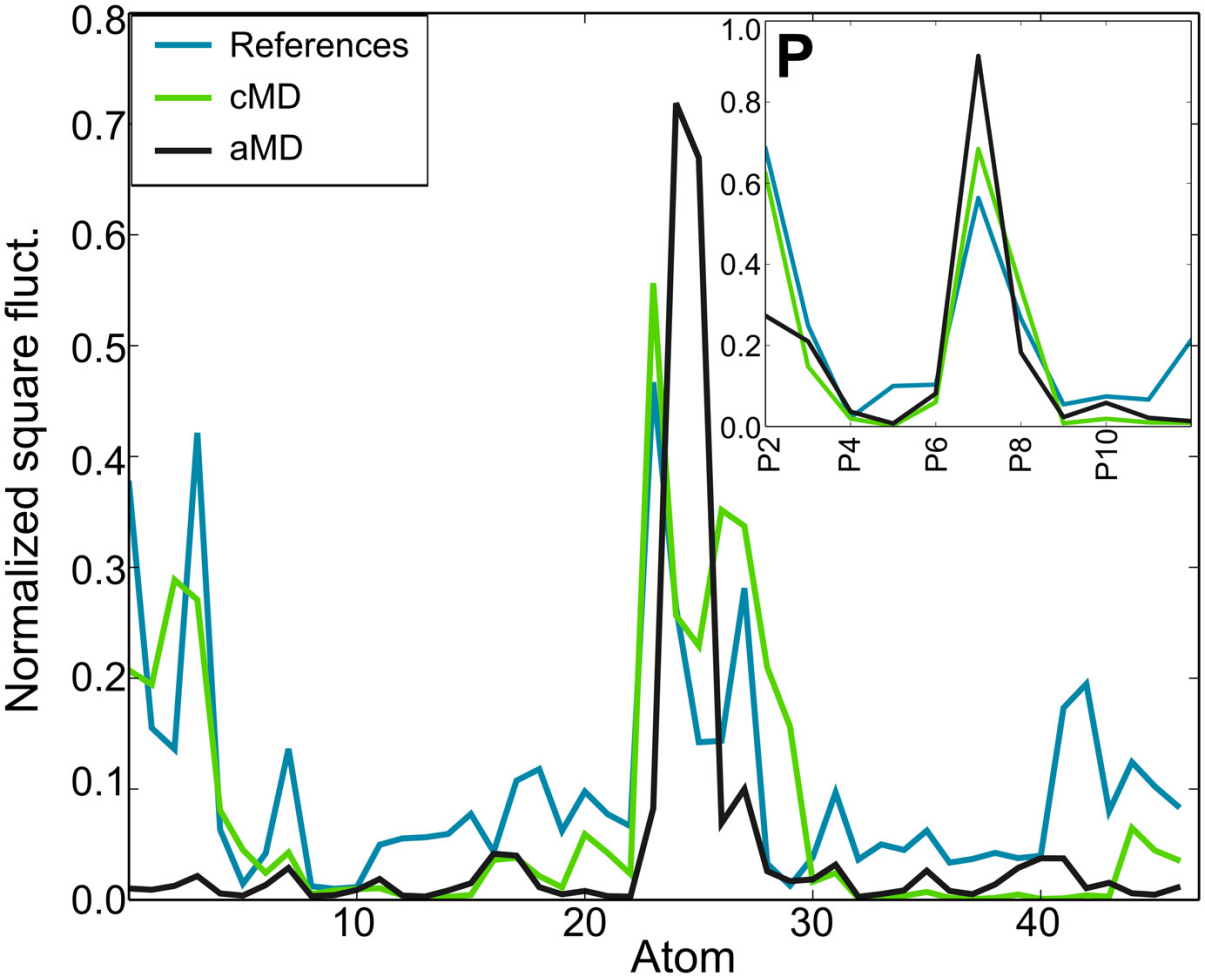
Atomic global fluctuations extracted from the *reference* structures, and the cMD and aMD ensembles. Only P, C2’, C4 and N3 atoms are considered. Computed fluctuations for the P atoms only are plotted in the box in the top right corner of the figure.

As we can see from Fig 6, the *references* global fluctuations are in qualitative agreement with the cMD simulation. Not surprisingly, aMD experiences the largest relative displacements. Due to limitations of the ANM models, interactions between beads depend on their distance and not the type of interaction, hence ANM fluctuations of the nucleobases are prone to be highly constrained. A differentiated correlation is observed when only backbone or nucleobase beads are considered. Fig 6 also shows a clear correlation between P-P fluctuations for each ensemble; thus, backbone global fluctuations are within the same range of native state fluctuations. On the other hand, no correlation exists when only N3 beads, which mainly represents the U-U configurational state, are considered due to a differentiated conformational exploration of the bases. Table 2 summarizes the Pearson correlation coefficients.

**Table 2:** Pearson correlation coefficients between CG set (P, C2’, C4 and N3), P beads and N3 beads fluctuations extracted from the *references*, cMD and aMD ensembles.

	CG	P	N3
**Ref | cMD**	**0.75**	**0.95**	0.34
**Ref | aMD**	0.28	**0.74**	0.11
**cMD | aMD**	0.37	**0.85**	-0.23

### Transferability of ANM to longer repeats

The transferability of ANM models optimized for short repeats to *n*-repeated structures is not only an interesting but also a controversial topic. Additional simulations were performed to investigate the effect of varying the length of CUG repeats in the reliability of MD and ANM models. Those were conducted with a 6-repeats rCUG (from a X-ray structure, PDB ID: 3gm7) and a 12-repeats rCUG (homology modeled). The essential subspace overlap between ANM and EDA modes extracted from the (CUG)_6_ accounts for a total of 47%, a 3% less than in the (CUG)_2_ fragment from the previous cMD simulation. However, the overall collectivity of PC1 increased up to 0.80 which reflected an even higher degree of cooperative dynamics. In contrast, the essential subspace overlap for (CUG)_12_ increased up to a 67%, but the collectivity degree remains similar to the (CUG)_6_ structure (0.73). The amount of residues involved in deformation movements was quite similar in both ANM and MD simulations, but there was a tendency of ANM to provide less collective fluctuations. As shown in Table 3, the ANM approach yielded an acceptable description of global flexibility in the crystal structure in terms of cumulative overlap of PC1 and PC2, using the 20 softest normal modes (> 50%). However, this value was substantially lower than those obtained for (CUG)_2_ and (CUG)_12_, and PC3 yields a poor overlap (38%). Interestingly, ANM performed better in predicting deformations from short and long CUG fragments. Unfortunately, the bias introduced by the simulation length is non-negligible and non-harmonic movements are likely to have more impact in simulations of short helices.

**Table 3:** Percentage of captured variance (%σ^2^), collectivity (κ) and cumulative overlap (CO) extracted from the first three PCs of models with 2, 6 and 12 CUG repeats. Cumulative overlap was calculated for the 20 softest normal modes.

	PCA	%σ^2^ (MD)	κ (MD)	κ (ANM)	%CO
**(CUG)**_**2**_	PC1	37.3	0.39	0.55	90
	PC2	22.4	0.63	0.40	72
	PC3	10.9	0.73	0.51	80
**(CUG)**_**6**_	PC1	30.3	0.80	0.76	52
	PC2	18.8	0.79	0.67	74
	PC3	15.7	0.75	0.46	38
**(CUG)**_**12**_	PC1	26.1	0.72	0.69	92
	PC2	20.2	0.71	0.73	60
	PC3	13.8	0.82	0.62	93

## Discussion

RNA plays critical roles in cellular biology hence it is an extremely important target for small molecule therapeutics. Unfortunately, knowledge of RNA-small molecule interactions is still scarce yet insights into the intricacies of the dynamics of RNA are essential to provide novel therapeutic scaffolds. In particular, targeting rCUG sequences has proved to be the most appropriate approach for treatment of DM1, since RNA acts as the causative agent, whereas MBNL1 remains unmodified. Structure-based drug design relies on understanding the target structure and its dynamic properties as the structural conformations are not unique in time. The rCUG are characterised by their ability to adopt a large set of conformations each of which has been observed in X-ray or NMR resolved structures. The druggability of non-canonical pairs is not yet well-established and their repertoire of possible conformations may increase the complexity of the system under study by including a large set of possibilities.[51,52]

In our study, we assessed two of the most relevant techniques to explore the rCUG conformational landscape, which are elastic network models (ENM) and molecular dynamics (MD). In agreement with previous studies, the use of ENM with a simplified coarse-grained representation is able to reproduce the global motions observed in experimental structures. In particular, the information gathered from the slow modes obtained from ANM is identical to that contained in the PDB ensemble. The best parameterization for our RNA model was obtained with a coarse-grained model represented by atoms P, C2’, C4 and N3. Optimal performance was achieved with a 9 Å cutoff and a distance dependent force constant. In this regard, we have confidence that ANM parametrization was able to correctly describe the global fluctuations of a highly dynamic RNA structure such as rCUG, but it required the consideration of both backbone and nucleotide coordinates to attain a good level of description.

In order to assess the viability of ANM methods to capture local motions derived from U-U pairs we proceeded to investigate their motions as described by MD simulations. MD is probably the most accurate computational method for the theoretical study of large-scale dynamics, since it is based on rigorous physical formalisms and quantum-mechanical and experimental parameterizations. However, its high computational cost still limits all-atom simulations to the microsecond scale. Comparison of cMD and aMD simulations demonstrated a clear difference in conformational space exploration. The softening of energy barriers that aMD provides allows to explore a higher number of internal loop conformations than cMD in the same time scale. For instance, the aMD trajectory analysis concluded that types I, III and V are preferred along the simulation. From the point of view of the rCUG local conformational landscape, the U-U pairs adopt preferentially a type IV conformation (1 hydrogen bond inclined towards the major groove) which is the most experimentally observed conformation.

Nonetheless, MD techniques allowed us to explore a higher myriad of conformations which induces high local fluctuations onto the structure. In line with these results, other studies have demonstrated that MBNL1 binding to U-U pairs induces local melting of the RNA structure. That recognition step depends on the occasional loss of the hydrogen bonding patterns of the internal loops. Nevertheless, both MD methods showed that the main motions, global and local, are highly dependent on the backbone heavy atoms displacement and the base pair opening effect. In addition, the local information is affected by the precision of the force field, which is compounded by inaccuracies in its parametrization. Thus, the conformational analysis is not void of errors and these can compromise the reliability of the data extrapolated from MD simulations. However, the short nanosecond time scale used in this study and the agreement in terms of overlapping global space with experimental structures permitted confident comparison of ENM and MD techniques. It is reasonable to assume that, even though we do not assess the intrinsic stability of the molecular system, a deformation along the ANM modes partially explores the conformational possibilities whereas force field parametrization of MD simulations guides the system through a more comprehensive conformational landscape. However, the harmonic approximation of this method requires a potential minimum, limiting the utility of ANMs to model non-equilibrium dynamics.

As mentioned above, ANM fail to properly describe local motions which can be easy discerned from the U-U pairing sampling. All U-U conformations found in the ANM ensemble correspond to type IV, the same as the ones in the *reference* structure. Therefore, the bulk of the results presented demonstrate that MD simulations are required if insights into U-U dynamics and transitions are relevant. In addition, U-U pairing highly depends on the ionic distribution and water mediated hydrogen bonds, which are not contained within the ANM formalism. Thus, the simplified potential of ANM cannot provide quantitative data about the intrinsic movements of CUG repeats.

From structure-based drug design point of view, traditional rigid docking fails to describe small molecule—RNA interactions due to the lack of RNA adaptation. In fact, a fast and practical approach to improving molecular docking in proteins is to generate an ensemble of conformations obtained from experimental structures. However, our results suggest that ANM are not suitable for structure-based drug because high local fluctuations are not efficiently captured. Several studies have succeeded in applying a molecular dynamics approach for drug-design of rCUG binders, [48,49] but time and computational limitations for exploring small molecule—RNA interactions exist and large virtual screening campaigns cannot be performed. Other authors have suggested the combined use of ENM and MD, [41] which can favour particular modes observed in the MD simulations over the global backbone motions, improving the reproduction of both local and global modes. In addition, all the metrics demonstrate the robustness of ANMs to reproduce harmonic global fluctuations observed during sub-microsecond MD simulations of different length RNAs.

In conclusion, our work gives a comprehensive comparative analysis of ANM and MD methods for assessing small scale and large scale events along a highly dynamic RNA structure. However, these results will be subject to improvements implemented in other RNA force fields, which are constantly being revised. Further analyses will be conducted to study and compare the accuracy of the force field revisions and their effect on the local and global configurations of the RNA. These studies should provide useful insights that could be exploited for computer-aided drug design strategies.

## Methods

### ANM/PCA/EDA from experiments and simulations

The system is represented by a set of nodes (one node per atom if all-atom representation; one or several nodes per residue if coarse-grained representation). The general form for the ANM harmonic potential is:

$$V_{ANM}=-\frac{\gamma }{2}\left[\overset{N-1}{\underset{i=1}{\sum }}\overset{N}{\underset{j=i+1}{\sum }}{\left(s_{ij}-s_{ij}^{0}\right)}^{2}\Gamma_{ij}\right]$$

where *s*_*ij*_ and *s*_*ij*_^*0*^ are the instantaneous and equilibrium distances of atoms *i* and *j* respectively, *γ* corresponds to a homogeneous force constant, and Γ_*ij*_ is the *ij*th element of the Kirchhoff matrix. In this study we tested several distance dependent γ weighted by a negative exponential function before finding the most suitable one (see Results section). The second order derivative of the potential energy function were collected in the Hessian matrix **H** which can be decomposed in 3*N*-6 nonzero *λ*_*i*_ eigenvalues and their corresponding eigenvectors ***u***^*i*^:

$$H=\overset{3N-6}{\underset{i=1}{\sum }}\lambda_{i}u^{i}u^{i}{}^{{}^{T}}$$

Principal modes were obtained by decomposing the covariance matrix (**C**) for the conformers:

$$C\;=\;\sum_{i\;=\;1}^{n}\sigma_{i}p^{i}{p^{i}}^{T}$$

where *σ*_*i*_ and *p*^*i*^ correspond to the *i*th eigenvalue and eigenvector of **C** respectively, and *n* is the number of non-zero eigenvalues. The ANM covariance matrix is directly related with Hessian matrix (i.e. **C**_**ANM**_ ∝ **H**^-1^), thus the PCA *σ*_*i*_ is the counterpart of 1/*λ*_*i*_ and ***u***^***i***^ is the counterpart of ***p***^*i*^.

For experimental validation of the ANM/PCA methods a (CUG)_3_ ensemble was constructed by aligning (CUG)_3_ fragments from available experimental structures (PDB IDs: 1zev [31], 3gm7 [29], 3syw [32], 3szx [32], 4e48 [30], 4fnj [33]). A total of 15 fragments were aligned with VMD and saved as a single rCUG PDB ensemble file. ANM analysis, which uses a single structure, were performed over one of the central CUG fragment of 3gm7. All ANM and PCA calculations were conducted with the ProDy suite.[53]

### Essential dynamics analysis (EDA)

Essential dynamics were based on the cross-correlation between the fluctuation of the P, C2’, C4 and N3 atoms observed during the molecular dynamics trajectory. Essential modes were obtained by decomposing the **C** matrix for 2000 equally distributed snapshots extracted from the simulations. Mean-square fluctuations were computed as previously described.[50]

### Comparison metrics for dominant modes

The overlap between ANM and PCA modes is given by the dot product of the corresponding eigenvectors:

$$O_{ij}\;=\;p^{i}\cdot u^{j}$$

The cumulative overlap was used to measure the correlation between predicted and experimental modes. The cumulative overlap is the extent to which a set of ANM soft modes can predict a PCA mode, hence it measures how well a subset of *J* ANM modes reproduces the *i*th PCA mode:

$$CO_{i}^{J}={\left[\overset{J}{\underset{j=1}{\sum }}{\left(O_{ij}\right)}^{2}\right]}^{1/2}$$

The essential subspace overlap between two subspaces spanned by top *K* modes is evaluated as:

$$SO^{K}={\left[\frac{1}{K}\overset{K}{\underset{i=1}{\sum }}\overset{K}{\underset{j=1}{\sum }}{\left(O_{ij}\right)}^{2}\right]}^{1/2}$$

The degree of collectivity (*κ*), which provides a measure of the extent of distribution of motions across the structure, was computed using the definition proposed by Brüschweiler [54]:

$$\kappa_{k}=\frac{1}{N}\text{exp}\left\{-\overset{N}{\underset{i=1}{\sum }}u_{ik}^{2}\text{log}\left(u_{ik}^{2}\right)\right\}$$

All these metrics were computed using the ProDy suite.[53]

### Models preparation for molecular dynamics

The molecular structure of a double stranded RNA with 2 CUG repeats in each slide was taken from high resolution X-ray data (PDB ID: 3gm7 [29]) and it was edited by capping the structural model with C•G pairs. The resulting model was prepared using *tleap* module from the Amber molecular dynamics package. We used the AMBER [55] force field with revised χ[56] and α/γ[57] torsional parameters for all simulations. A total of 14 Na^+^ counterions were added to neutralize the system charge using Joung and Cheatham parameters.[58] The system was solvated in a truncated octahedron with a spacing of 12 Å around the RNA using the TIP3P [59] water model. (CUG)_6_ model was prepared as previously described using the X-ray 3gm7 structure. (CUG)_12_ model was prepared with *tleap* using 3gm7 model as template.

### Conventional molecular dynamics protocol

Prior to the production phase each system was prepared using the following protocol: the RNA was constrained and the solvent and counterions were minimized during a 2,500-step minimization stage. Next, a second minimization 15,000-step long simulation was run without constrains. After system minimization, the backbone was constrained and the system was heated at 300 K within 100 ps followed by a 200 ps long MD at 300 K with decreasing force constraints. After relaxation, a 2 ns long MD was performed under N*p*T ensemble (*p* = 1 bar, T = 300 K) allowing density balance. The production trajectories were obtained at NVT conditions at 300 K. In all the simulations the PME [60,61] method for treatment of electrostatic interaction was used under periodic boundary conditions. A 9 Å short-range cutoff was applied and the SHAKE [62] algorithm was used to fix all hydrogen atom positions. The time step was fixed to 2 fs and coordinates were stored every 1 ps. The total simulation time was 200 ns.

### Accelerated molecular dynamics protocol

Accelerated molecular dynamics (aMD) simulations were conducted using the same protocol as equilibration and production runs of conventional molecular dynamics. The first 30 ns of the previous production trajectory were used to calculate the parameters related to the average total potential energy and the average dihedral energy parameters, required for aMD parametrization (i.e. EthreshD = 483 kcal/mol, alphaD = 11 kcal/mol, EthreshP = -36257 kcal/mol, alphaP = 2192 kcal/mol).

### Molecular dynamics analysis

Conformations and frequencies of the different non-canonical U-U pairs were analyzed on each trajectory using the clustering tool from Amber *cpptraj*[55] module. Clustering was performed over each non-canonical U-U pair using the average-linkage algorithm and specifying a minimum distance of 3.0 Å.

Helical parameters were monitored using the 3DNA [63] software and extracted at intervals of 20 ps, including intra-base pair parameters (shear, stretch, stagger, buckle, propeller and opening), inter-base pair parameters (shift, slide, rise, tilt, roll and twist) and backbone torsions.

## Supporting Information

S1 Fig Maximum overlapping achieved by using different cutoffs and residues description: all-atom, CG-1 (atoms P, C2’, C4’) and CG-2 (atoms P, C2’, C4’, N3). A similar overlapping is achieved with an all-atom model and r_c_ = 14 and CG-2 and r_c_ = 9.
(TIFF)

S2 Fig Base pair (shear, stretch, stagger, buckle, propeller and opening) and base step parameters (shift, slide, rise, tilt, roll and twist) extracted from the cMD simulation. Average and standard deviation are indicated.
(TIFF)

S3 Fig Base pair (shear, stretch, stagger, buckle, propeller and opening) and base step parameters (shift, slide, rise, tilt, roll and twist) extracted from the aMD simulation. Average and standard deviation are indicated.
(TIFF)

S4 Fig RMSD of the cMD (black lines) and aMD (blue line) simulations. (TIFF)

S5 Fig Graphical representation of the three RNA structures considered in the study. The effect of varying the length was evaluated using 2, 6 and 12-repeats rCUG.
(TIFF)

S1 Table Structural parameters inferred from MD simulation clustering (CUG centroids) and experimental data. The same tabular format as Coonrod et al. has been used for comparison purposes (Coonrod et al. (2012). Biochemistry, 51, 8330–37).
(PDF)
