# Supplementary material for: On the Applicability of Elastic Network Models for the Study of RNA CUG Trinucleotide Repeat Overexpansion
Source: PLoS One. 2016 Mar 24;11(3):e0152049. doi: 10.1371/journal.pone.0152049 (PMC4806922; doi:10.1371/journal.pone.0152049)
Supplement: S1 Table — The same tabular format as Coonrod et al. has been used for comparison purposes (Coonrod et al. (2012). Biochemistry, 51, 8330–37). (PDF) [file pone.0152049.s006.pdf]

S1 Table. Structural parameters inferred from MD simulation clustering (CUG centroids) and experimental data. The same tabular format as Coonrod *et al*. has been used for comparison purposes (Coonrod et al. (2012). Biochemistry, 51, 8330–37).

Structure	Pair	C1'-C1' (Å)	1st HB (Å)	2nd HB (Å)	λI (°)	λII (°)	Incline	Shear (Å)	Stretch (Å)	Stagger (Å)	Buckle (°)	Propeller (°)	Opening (°)
**CUG centroids**	U3-U14	10,2	2.9	-	67,5	31,4	Major	3.10	-1.53	-0.05	-2.92	-10.39	-11.58
	U3-U14	10,9	2.7	-	25,2	57,4	Minor	-2.98	-1.14	0.18	3.87	-5.26	-27.76
	U6-U11	10,9	2.8	-	20,4	62,6	Minor	3.37	-1.72	-0.05	-1.68	-1.60	-15
	U6-U11	10,1	2.8	-	70,6	30	Major	-3.33	-1.36	-0.67	1.35	-13.86	-13.93
**Disney NMR**	U5-U14 (0)	10.7	-	-	71.2	32	Major	3.57	-0.95	-0.05	13.48	-9.09	-5.62
	U5-U14 (1)	10.6	2.9	-	61.1	28.9	Major	3.03	-1.14	-0.09	-22.07	-6.46	-24.3
	U5-U14 (2)	8.9	2.9	2.9	73.3	48.3	Major	2.24	-1.74	0.02	12.56	-17.25	4.58
**Kiliszek *et al.***	U3-U6 (A-B)	10.7	2.8	-	26.2	57.9	Minor	-2.82	-1.19	-0.47	12.51	-8.17	-31.5
	U6-U3 (A-B)	10.3	2.9	-	31.6	67.7	Minor	-3.03	-1.31	-0.13	14.01	-8.36	-16.37
	U3-U6 (C-D)	10.9	2.8	-	56.1	22.6	Major	2.98	-1.22	-0.44	-4.51	-8.69	-34.42
	U6-U3 (C-D)	10.2	2.6	-	59.2	32.4	Major	2.59	-1.51	-0.28	0.57	-15.81	-21.16
	U3-U6 (E-E*)	10.6	2.6	-	52.7	29.1	Major	2.38	-1.31	-0.48	-4.07	-11.84	-30.56
	U6-U3 (E-E*)	10.6	2.6	-	29.1	52.7	Minor	-2.38	-1.31	-0.48	4.07	-11.84	-30.56
